# Lysosomal Dysfunction Promotes Cleavage and Neurotoxicity of Tau *In Vivo*

**DOI:** 10.1371/journal.pgen.1001026

**Published:** 2010-07-15

**Authors:** Vikram Khurana, Ilan Elson-Schwab, Tudor A. Fulga, Katherine A. Sharp, Carin A. Loewen, Erin Mulkearns, Jaana Tyynelä, Clemens R. Scherzer, Mel B. Feany

**Affiliations:** 1 Department of Pathology, Brigham and Women's Hospital and Harvard Medical School, Boston, Massachusetts, United States of America; 2 Department of Neurology, Brigham and Women's Hospital and Harvard Medical School, Boston, Massachusetts, United States of America; 3 Whitehead Institute for Biomedical Research, Cambridge, Massachusetts, United States of America; 4 Department of Cell Biology, Harvard Medical School, Boston, Massachusetts, United States of America; 5 Institute of Biomedicine/Medical Biochemistry and Developmental Biology, Helsinki University, Helsinki, Finland; Thomas Jefferson University, United States of America

## Abstract

Expansion of the lysosomal system, including cathepsin D upregulation, is an early and prominent finding in Alzheimer's disease brain. Cell culture studies, however, have provided differing perspectives on the lysosomal connection to Alzheimer's disease, including both protective and detrimental influences. We sought to clarify and molecularly define the connection *in vivo* in a genetically tractable model organism. Cathepsin D is upregulated with age in a *Drosophila* model of Alzheimer's disease and related tauopathies. Genetic analysis reveals that cathepsin D plays a neuroprotective role because genetic ablation of cathepsin D markedly potentiates tau-induced neurotoxicity. Further, generation of a C-terminally truncated form of tau found in Alzheimer's disease patients is significantly increased in the absence of cathepsin D. We show that truncated tau has markedly increased neurotoxicity, while solubility of truncated tau is decreased. Importantly, the toxicity of truncated tau is not affected by removal of cathepsin D, providing genetic evidence that modulation of neurotoxicity by cathepsin D is mediated through C-terminal cleavage of tau. We demonstrate that removing cathepsin D in adult postmitotic neurons leads to aberrant lysosomal expansion and caspase activation *in vivo*, suggesting a mechanism for C-terminal truncation of tau. We also demonstrate that both cathepsin D knockout mice and cathepsin D–deficient sheep show abnormal C-terminal truncation of tau and accompanying caspase activation. Thus, caspase cleavage of tau may be a molecular mechanism through which lysosomal dysfunction and neurodegeneration are causally linked in Alzheimer's disease.

## Author Summary

Neurodegenerative disorders, like Alzheimer's disease, are a devastating group of conditions that exact a heavy toll on patients and their families. These disorders also represent a significant and growing public health challenge as our population ages because no effective treatments are available. Research over the past two decades has strongly suggested that a fundamental problem in affected nerve cells relates to accumulation of cellular “garbage,” or proteins and other material that is too old to function properly. Thus, understanding how the neuron handles these outdated molecules is of great significance. Here we find that upregulation of one such cellular degrading pathway, the lysosome, can have significant deleterious effects to the neuron. We specifically show that expanding the lysosomal compartment can markedly increase production of a very toxic form of tau, a protein strongly implicated in neuronal dysfunction and death in Alzheimer's disease and related disorders. Our findings have important implications for the development of neurodegenerative disease therapies that seek to manipulate the lysosome and the proteins within the lysosome.

## Introduction

Tauopathies, including Alzheimer's disease, are characterized by abnormal intraneuronal accumulation of the microtubule-associated protein tau. The identification of dominant mutations in the gene encoding tau in hereditary tauopathies (frontotemporal dementia with Parkinsonism linked to chromosome 17; FTDP-17) has established a causal relationship between tau and neurodegeneration. Interestingly, lysosomal alterations also accompany these complex neurodegenerative disorders. Lysosomes are responsible for autophagy, mediating proteolysis and recycling of cellular and endocytosed materials, and their degradative activities depend critically upon the cathepsin proteases [1]. Application of anti-cathepsin antibodies to Alzheimer's disease brain has revealed widespread expansion of the lysosomal system early in Alzheimer's disease. The increased numbers and density of structurally abnormal lysosomes found in Alzheimer's disease neurons is specific for affected brain regions [2], [3]. Similar changes occur in Alzheimer's disease rodent models, including mice in which mutant tau is overexpressed [4].

Striking loss of neurons in a number of inherited, predominantly pediatric lysosomal storage disorders reveals neuronal vulnerability to lysosomal dysfunction [1]. However, the role of lysosomal abnormalities in adult neurodegenerative disorders, including tauopathies and Alzheimer's disease, remains unclear. Broadly, there are three distinct, although not entirely mutually exclusive, views. First, defective lysosomal function could lead to accumulation of the proteins implicated in pathologic Alzheimer's disease aggregates, beta-amyloid and tau, through abnormal proteolytic processing [3]. Lysosomal proteases have been shown, for example, to degrade tau *in vitro* and in cultured cells [5], [6]. Second, lysosomal dysfunction could indirectly promote neurodegeneration through abnormal permeabilization of the lysosome. Decreased stability of lysosomal membranes occurs downstream of a variety of cytotoxic stressors and leads to the release of lysosomal contents and caspase-dependent apoptosis [7]. Alternatively, it has been suggested that lysosomal expansion could be a cytoprotective response in Alzheimer's disease, directly facilitating the degradation of toxic proteins via autophagy [8].

Of the widespread lysosomal abnormalities described in Alzheimer's disease, there is conspicuous upregulation of mRNA and protein of the aspartyl protease cathepsin D within degenerating neurons [9], [10]. Polymorphisms in the cathepsin D-encoding gene *CTSD* have also been associated with Alzheimer's disease [11], [12]. The discovery that mutations in cathepsin D cause aggressive neurodegeneration in one form of neuronal ceroid lipofuscinosis, a childhood neurodegenerative lysososomal storage disease [13], [14] has suggested upregulation in Alzheimer's disease may be a compensatory protective response. In contrast, other studies have suggested that cathepsin D can act a pro-apoptotic molecule [15]. Still other work suggests a direct relationship between tau and cathepsins, showing cathepsin inhibition promotes tau accumulation and post-translational modifications including phosphorylation [16].

To investigate the role of lysosomal expansion and cathepsin D in tauopathies, we employed a genetically tractable *in vivo* approach. As in Alzheimer's disease, microarray analysis of a well-characterized *Drosophila* model of tauopathy identified lysosomal dysfunction and cathepsin D upregulation. Genetic ablation of cathepsin D caused lysosomal expansion and caspase activation, and markedly potentiated tau-induced toxicity. The latter effect depended upon caspase-directed cleavage of tau that generated a toxic C-terminally truncated tau species. Furthermore, cathepsin D deficiency in mice and sheep also led to caspase activation and C-terminal truncation of tau. Our results suggest that caspase cleavage of tau is a molecular target of lysososomal dysfunction in Alzheimer's disease and possibly other human neurodegenerative disorders.

## Results

### Removing cathepsin D specifically enhances tau-induced neurodegeneration and cell-cycle activation

**Figure 1:**
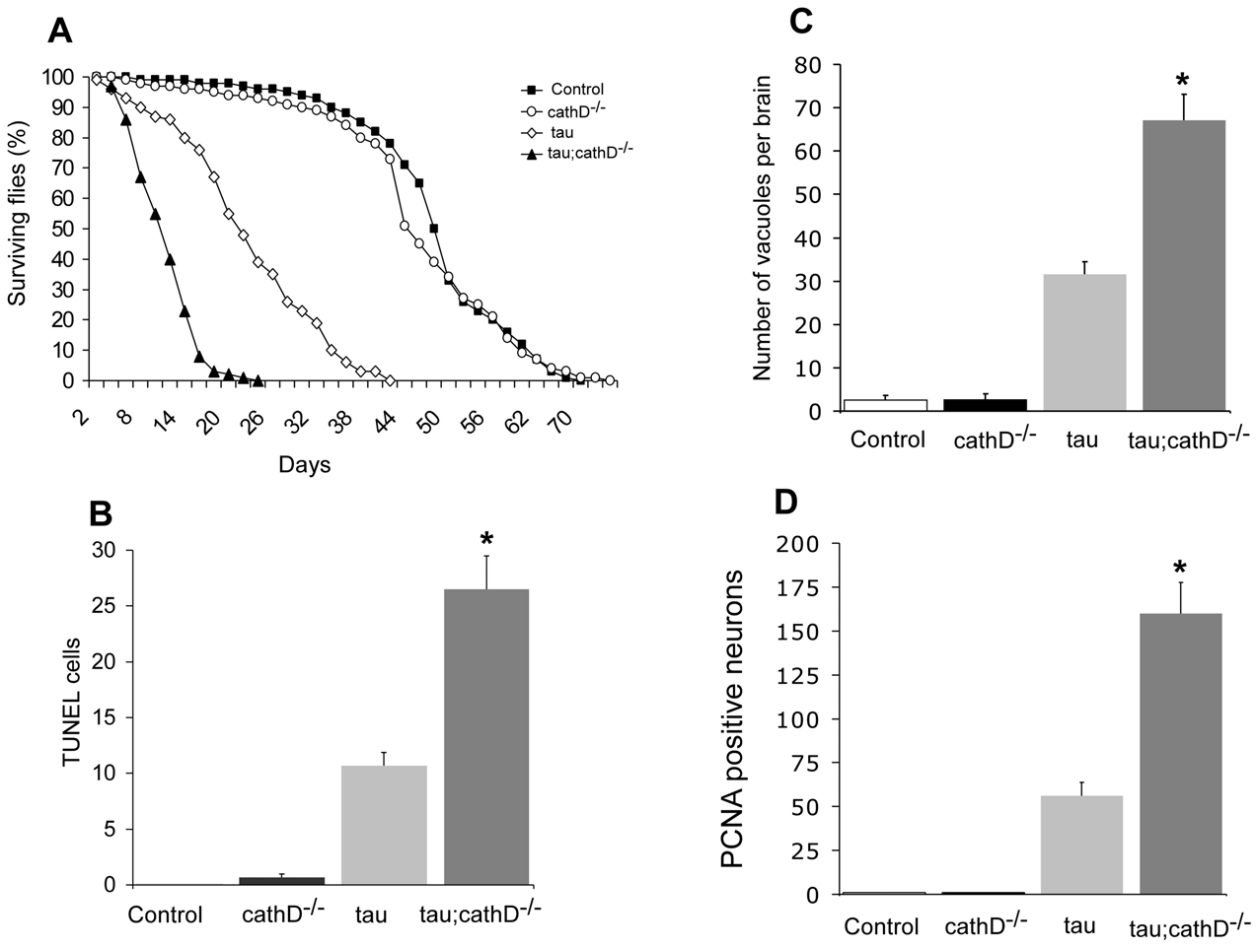
Neurotoxicity of human tau is markedly increased in the absence of cathepsin D. (A) Lifespan of tau transgenic flies is shortened in a *cathepsin D* null background. (B,C) Neurodegeneration, as indicated by the number of TUNEL-positive cells (B) or vacuoles (C) is significantly increased in tau transgenic flies in a *cathepsin D* null background. (D) Cell cycle activation, as indicated by PCNA immunostaining, is significantly increased by removing cathepsin D. *indicates P<0.01, one-way ANOVA with Student-Neuman-Keuls post hoc test for multiple comparisons. Flies are 10 days old. Control is *elav-GAL4/+* in all panels.

In flies, neuronal expression of human tau, whether wild-type or mutant forms linked to hereditary tauopathies (FTDP-17), causes apoptotic neurodegeneration with brain vacuolization and TUNEL labeling of neurons [17], [18], [19]. Expression of mutant tau^R406W^ induces a level of toxicity well suited for genetic analysis [17], [18], [20]. The R406W mutant form of tau is thus used primarily in the current paper, and is termed “tau” for simplicity. In an unbiased, genome-wide expression analysis of aging tau transgenic flies, we previously observed significant changes in the expression of lysosomal genes suggestive of general lysosomal dysfunction in our model [21]. Cathepsins, including cathepsin D, are lysosomal enzymes that are evolutionarily conserved from *Drosophila* to humans [22]. Here we confirmed prominent cathepsin D overexpression in the tauopathy model at early and advanced disease stages compared to age-matched control animals using quantitative PCR (fold-change, 1.6 with *P* = 0.005, and 2 with *P* = 0.037, respectively; Figure S1), thus recapitulating findings from Alzheimer's disease patients. These data provide a rationale for determining the effect of genetically modulating cathepsin D levels on tau-induced neurodegeneration. Conveniently, complete removal of cathepsin D in flies is not lethal [22]. Tau transgenic flies were accordingly crossed into a cathepsin D null background. As previously reported, flies overexpressing tau exhibit reduced lifespan compared to control animals [19]. Removing cathepsin D further reduced the longevity of tau transgenic flies by almost 50%, while deletion of cathepsin D on a wild-type background had no clear effect (Figure 1A).

To establish whether the decrease in lifespan correlated with increased neurotoxicity, we determined the degree of apoptotic cell death by counting the number of TUNEL-positive neurons in brain sections. As previously described, non-transgenic and cathepsin D null control flies exhibited negligible TUNEL-positivity at 10 days [22], while neuronal death in tau transgenic flies was detected by TUNEL staining [18] (Figure 1B). Consistent with the lifespan data, flies expressing tau in a cathepsin D null mutant background showed an almost 3-fold increase in apoptotic cell death (Figure 1B). A similar effect on neurodegeneration was revealed by histological analysis and subsequent quantification of vacuolar degeneration in aged fly brains (Figure 1C). Together, these data suggest reduction in cathepsin D significantly promotes tau-induced neurodegeneration *in vivo*.

We have previously shown that tau-induced cell cycle reentry mediates neuronal apoptosis in the fly tauopathy model [18], a result that has been corroborated in rodent models model of tauopathy [23], [24]. To test whether removal of cathepsin D enhanced tau-induced neurodegeneration through abnormal cell-cycle activation, we immunostained brains from 10-day-old flies for proliferating cell nuclear antigen (PCNA), an S-phase cell-cycle marker abnormally upregulated in brains of Alzheimer's disease patients and tau-expressing flies. As for TUNEL, removal of cathepsin D markedly increased PCNA staining (approximately 3 fold) in flies expressing tau in cathepsin D-deficient genetic background (Figure 1D). Non-transgenic and cathepsin D null control flies were immunonegative for PCNA. These results suggest that cathepsin D modulation of tau toxicity occurs via abnormal cell-cycle re-entry.

The human disease literature suggests that early lysosomal abnormalities, including cathepsin D upregulation, are particularly prominent in Alzheimer's disease compared to other adult-onset neurodegenerative disorders [3]. We have previously characterized degeneration of postmitotic neurons in a fly model of Machado-Joseph disease (spinocerebellar ataxia type 3), an autosomal dominant neurodegenerative disease caused by polyglutamine expansion within the protein ataxin 3. Panneural expression of mutant human ataxin 3 in *Drosophila* causes age-dependent neurodegeneration [25], [26], as quantified by decreased numbers of Kenyon cells in the mushroom body [25]. Removing cathepsin D from mutant ataxin 3 overexpressing flies had no effect on Kenyon cell loss (Figure S2), suggesting that removal of cathepsin D is not generally toxic in neurodegenerative disease models.

### Removal of cathepsin D does not alter total tau levels or tau phosphorylation

**Figure 2:**
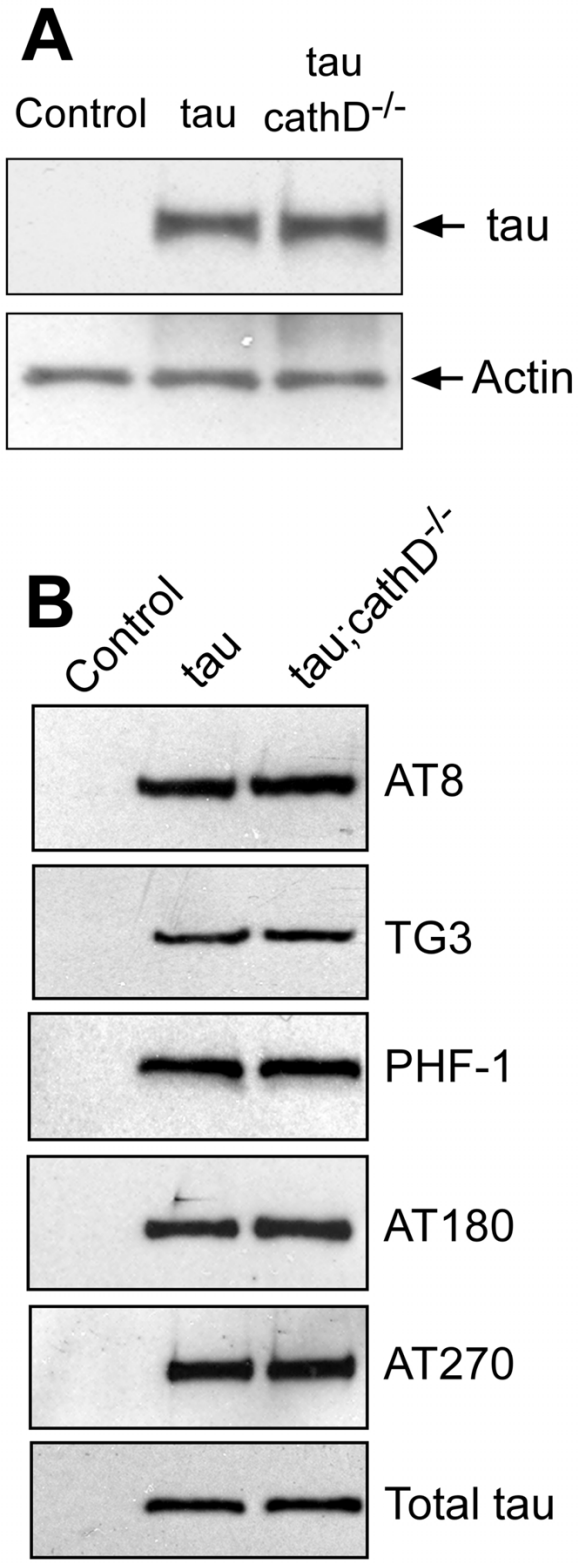
Removing cathepsin D does not substantially alter levels of tau. (A) No clear alteration in the levels of tau as monitored by a phosphorylation-independent antibody in *cathepsin D* mutant animals. The blot was reprobed for actin to document equivalent protein loading (bottom panel). (B) No clear changes in levels of tau phosphorylated at a variety of disease-associated residues in the absence of cathepsin D. The blot was reprobed with a phosphorylation-independent anti-tau antibody to ensure equivalent loading (bottom panel, Total tau). Flies are 10 days old. Control is *elav-GAL4/+*.

Since cathepsins have implicated in tau degradation, reduction of cathepsin D could potentiate tau toxicity by increasing total levels of tau. However, Western blots did not reveal an increase in tau levels in our model (Figure 2A). We next looked at potential post-translational modifications of tau that promote neurodegeneration. Tau hyperphosphorylation is a common post-translational modification in human tauopathies, and tau phosphorylation promotes neurodegeneration in *Drosophila* [27], [28], [29]. Analysis of adult fly head homogenates by Western blotting did not, however, reveal any appreciable change in tau phosphorylation at a number of disease-associated phosphoepitopes (AT8, TG3, PHF-1, AT180, AT270; Figure 2B). Enhancement of tau-induced neurodegeneration observed in cathepsin D-deficient flies thus did not appear to be mediated by an increase in total tau levels or tau phosphorylation.

### Removal of cathepsin D increases caspase-cleaved tau, which colocalizes with activated caspase-3

**Figure 3:**
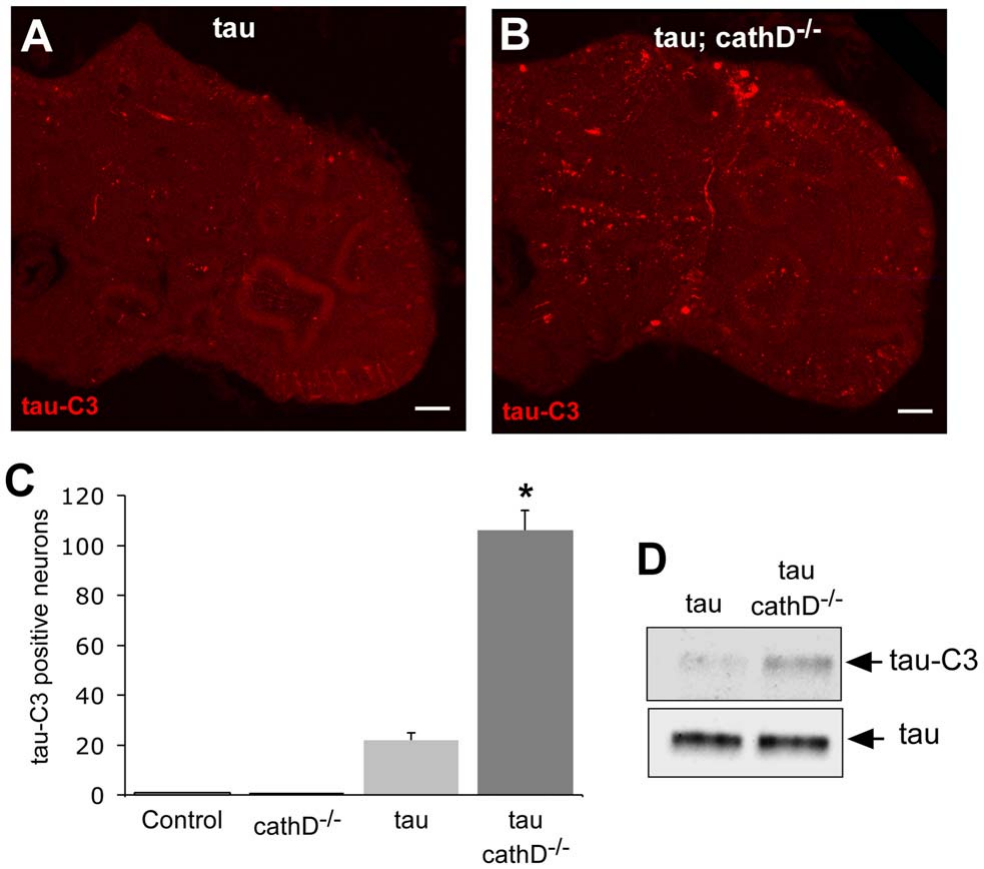
C-terminally truncated tau is elevated when cathepsin D is absent. (A,B) Increased levels of C-terminally truncated tau as detected by immunofluorescence in sections of fly brains from tau transgenic animals (B), as compared with controls (A) using the monoclonal antibody tau-C3, which specifically recognizes tau truncated at D421. Scale bars are 20 µm. (C) Quantitative analysis of immunostained sections reveals a signicant increase in the number of neurons containing truncated tau. *indicates P<0.01, one-way ANOVA with Student-Neuman-Keuls post hoc test for multiple comparisons. (D) Immunoblotting using the tau-C3 antibody to recognize truncated tau also demonstrates increased levels of truncated tau in *cathepsin D* mutant tau transgenic flies. The blot was reprobed with a polyclonal antibody recognizing tau to ensure equivalent loading (bottom panel, tau). Flies are 10 days old. Control is *elav-GAL4/+*.

The pharmacologic inhibition of cathepsins in hippocampal slice culture has been shown to promote cytosolic proteolysis of tau [30], Cleavage at one particular proteolytic site, the ^418^DXXD^421^ canonical caspase site, has been shown to occur in human tauopathies [31], [32], [33]. Caspase-3 is one of the key executioners of apoptosis in mammals, with homologous proteases in *Drosophila* including Drice and DCP-1. The caspase 3-cleaved form of tau, truncated at D421, is toxic in cell culture [34], [35], [36] and fibrillogenic *in vitro* [31], [33]. To address whether genetic loss of cathepsin D promotes tau proteolysis in transgenic flies, levels of truncated tau were analyzed using a monoclonal antibody specific to caspase cleaved tau, tau-C3 [31]. The scattered tau-C3-immunopositive staining observed in brain sections from transgenic tau flies was increased approximately 5-fold in the absence of cathepsin D (Figure 3A–3C, Figure S3). Western blot confirmed the elevation in C-terminally truncated tau in cathepsin D mutants (Figure 3D, Figure S4).

**Figure 4:**
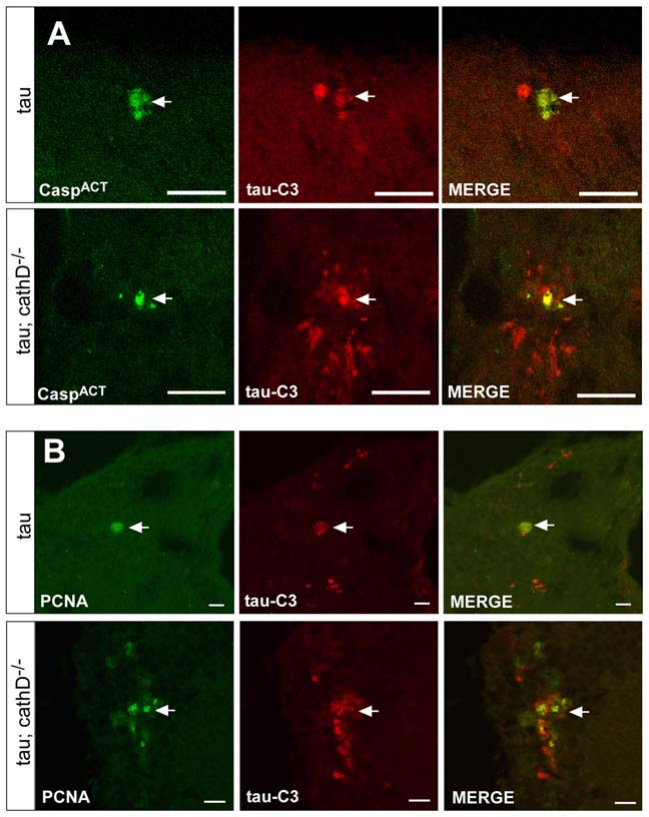
Truncated tau co-localizes with markers of cellular injury. (A) Immunoreactivity for truncated tau as detected with the tau-C3 monoclonal antibody (red) colocalizes with activated caspase (green). Arrows indicate colocalization. (B) Truncated tau (tau-C3, red) is found in neurons also expressing the cell cycle marker PCNA (green). Arrows indicate colocalization. Flies are 10 days old.

To determine if tau truncation could be linked to caspase activation, we used an antibody recognizing cleaved caspase-3 to assess caspase activation in fly brains. This antibody detects a fragment of activated caspase-3, and also labels dying cells in *Drosophila* where it may specifically recognize the effector caspase Drice [37], [38]. We used the antibody to confirm the presence of activated caspase-positive neurons in the brains of tau transgenic flies in the presence of absence of cathepsin D. Activated caspase colocalized with tau-C3 staining, consistent with caspase activation underlying the increased tau-C3 staining in cathepsin D-deficient tau transgenic animals (Figure 4A). No immunoreactivity for activated caspase was observed in control animals (*elav-GAL4/+*, 10 days-of-age; data not shown). We have previously demonstrated that tau-induced cell-cycle activation is downstream of tau phosphorylation and directly precedes apoptosis [18]. Interestingly, we also observed significant colocalization between tau-C3 and PCNA (Figure 4B). Control fly brains (*elav-GAL4/+*, 10 days-of-age) do not show expression of PCNA [17], [18]. The aberrant activation of cell cycle in neurons containing truncated tau strengthens the linkage between caspase-directed cleavage of tau and neuronal degeneration through abnormal cell cycle activation.

### Caspase-cleaved tau has increased toxicity, which is not altered by removing cathepsin D

**Figure 5:**
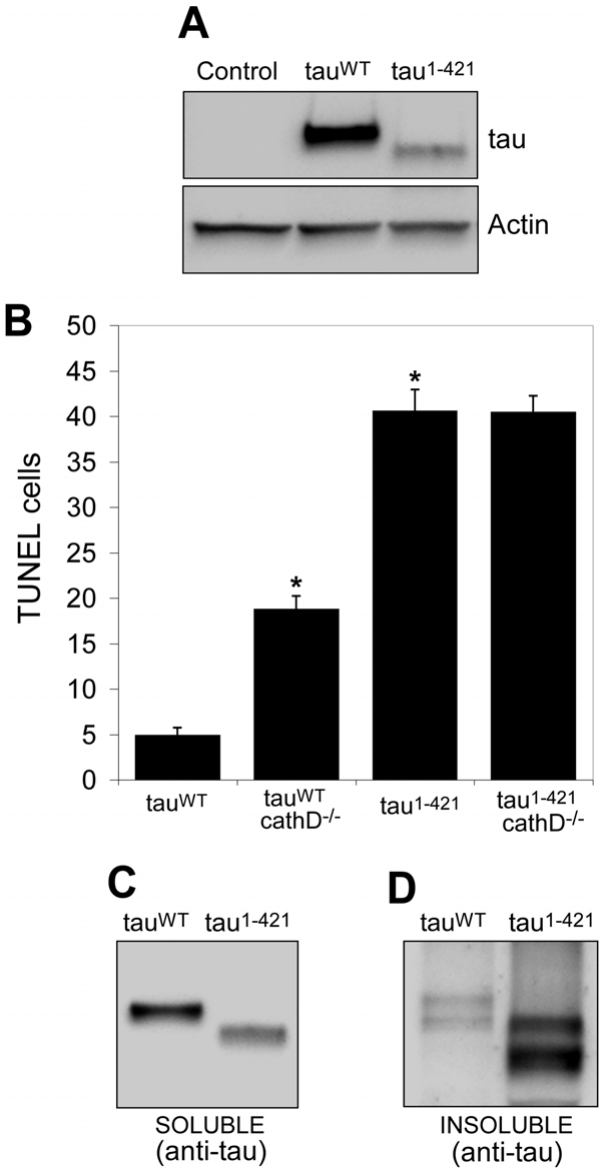
Truncation of tau significantly increases neurotoxicity and decreases solubility *in vivo*. (A) Lower levels of tau^1–421^ compared to tau^WT^. The blot was reprobed for actin to document equivalent protein loading (bottom panel). Control is *elav-GAL4/+*. (B) Neurodegeneration, as indicated by the number of TUNEL-positive cells, is significantly elevated tau^1–421^ in transgenic flies compared to tau^WT^, despite expression at lower levels. Removing cathepsin D significantly enhances the toxicity of tau^WT^, but not of tau^1–421^. *indicates P<0.01, one-way ANOVA with Student-Neuman-Keuls post hoc test for multiple comparisons. (C,D) Solubility of tau^1–421^ is reduced compared to tau^WT^, as evidenced by preferential recovery of tau^1–421^ in the insoluble fraction (D) following detergent solubilization and differential centrifugation. Flies are 10 days old.

Given the robust increase in C-terminally truncated tau following removal of cathepsin D, we characterized the effects of C-terminal truncation *in vivo*. The fly tauopathy model provides an opportunity to compare directly the toxicity of different forms of tau *in vivo*. We thus generated a transgenic *Drosophila* line expressing wild-type human tau truncated at Asp-421 (tau^1–421^) and compared the effect of its expression in the fly brain to that of full-length wild-type tau (tau^WT^). We chose to examine truncation of tau in the context of the wild-type protein, rather than the FTDP-17 linked R406W form of tau previously used in the paper because truncation of tau has been most extensively characterized in the context of wild-type tau [31], [32], [33]. The highest expressing lines of tau^1–421^ we recovered expressed significantly less tau compared to our tau^WT^ control lines (Figure 5A). Serial dilution experiments revealed that tau^1–421^ was expressed at approximately 20% of tau^WT^ levels (data not shown). Nonetheless, the number of TUNEL-positive neurons in the brains of flies expressing tau^1–421^ was significantly higher than in tau^WT^-expressing flies (by approximately 8 fold; compared number of TUNEL-positive cells in Figure 5B), establishing that caspase-cleaved tau is considerably more toxic *in vivo* than wild-type tau. Just as we showed for tau^R406W^, removal of cathepsin D potentiated the toxicity of tau^wt^ (by approximately 4 fold). Unlike wild-type tau, however, removing cathepsin D did not enhance the toxicity of tau^1–421^ (Figure 5B). The inability of cathepsin D removal to alter the toxicity of caspase-cleaved tau places C-terminal cleavage of tau downstream of cathepsin D.

Previous studies have demonstrated that C-terminal truncation of tau promotes fibrillization *in vitro* [31], [39], [40]. We therefore assessed the solubility of tau in our *in vivo* system. Consistent with the *in vitro* studies, we found a substantially higher fraction of nonionic detergent-insoluble tau in the brains of tau^1–421^ animals compared to tau^WT^ transgenics (Figure 5C and 5D, Figure S5, Figure S6). Together with the finding of increased C-terminally truncated tau in the cathepsin D null background (Figure 3), our data mechanistically link the formation of the less soluble and considerably more toxic C-terminally truncated form of tau to modulation of neurotoxicity by cathepsin D.

### Loss of cathepsin D results in caspase-dependent cleavage of PARP and expansion of the lysosomal compartment

**Figure 6:**
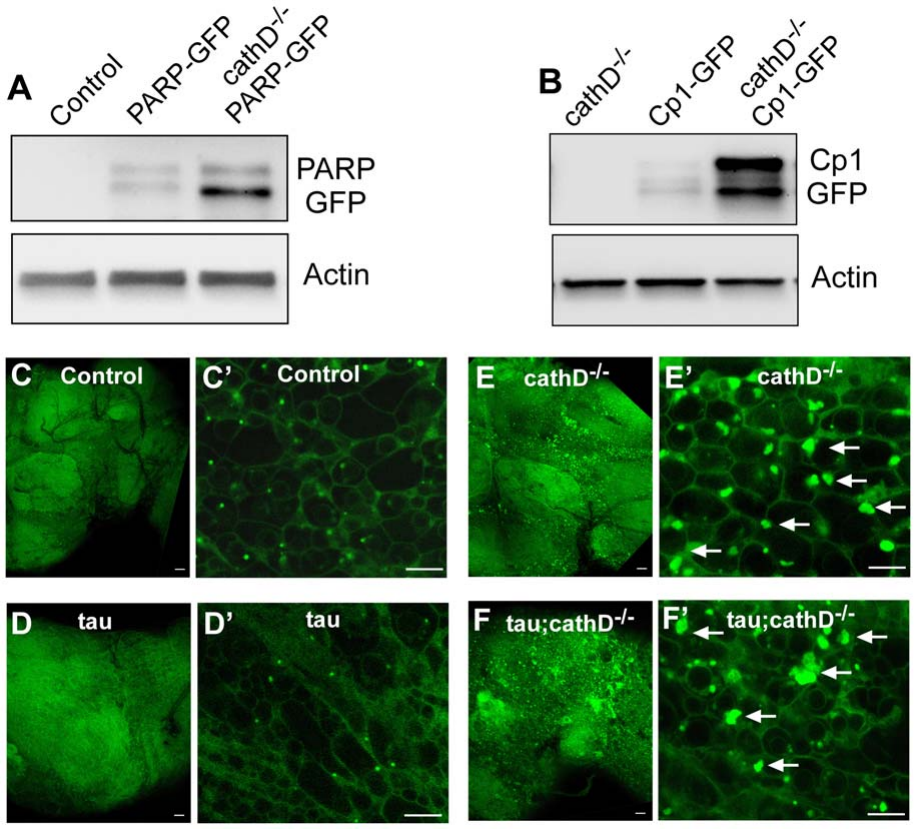
Widespread lysosomal abnormalities in cathepsin D deficient brains. (A) Cleavage of a transgenic PARP-GFP fusion protein is increased in *cathepsin D* mutant flies as revealed by immunoblotting with an anti-GFP antibody. The blot was reprobed for actin to document equivalent protein loading (bottom panel). (B) The levels of a GFP tagged lysosomal protease, Cp1-GFP, are markedly increased in *cathepsin D* mutants, as assessed by immunoblotting with an anti-GFP antibody. The blot was reprobed for actin to document equivalent protein loading (bottom panel). (C-F) Lysotracker staining confirms widespread expansion of the lysosomal system in cathepsin D deficient brains (E,F) compared with controls (C,D). Enlargement of lysosomes can be appreciated at higher power (C′–F′, arrows). No clear abnormalities in lysosomes were produced by the expression of tau in the presence (D,D′) or absence (F,F′) of cathepsin D. Scale bars in (C–F) are 20 µm. Scale bars in (C′–F′) are 5 µm. Control is *elav-GAL4/+* in all panels. Flies are 10 days old.

Our data suggested that loss of cathepsin D potentiates tau-induced neurotoxicity via caspase-dependent C-terminal truncation of tau (Figure 3, Figure 4). To further probe activation of caspase in our system we employed a genetically encoded reporter construct. Transgenic flies have been created that express a caspase substrate, human poly-ADP-ribose polymerase-1 (PARP). Human PARP is cleaved by mammalian caspase-3 and by homologous *Drosophila* effector caspases. The construct contains 40 amino acids of PARP, including the caspase cleavage site, and the Venus reporter that can be recognized by an antibody to GFP [41]. The smaller cleaved product can be detected by mobility shift on Western blot. Utilizing this reporter, we found a significant increase in cleaved PARP in fly brains after genetic deletion of cathepsin D (Figure 6A), strongly suggesting that reducing cathepsin D levels led to caspase activation.

We next investigated the mechanism through which removal of cathepsin D leads to caspase activation. A number of studies have shown that lysosomal abnormalities can lead to destabilization and increased permeability of the lysosomal membrane. Lysosomal proteases then activate a proteolytic cascade leading to caspase activation and apoptosis [7], [15]. We have previously shown accumulation of lysosomal storage material in cathepsin D null flies [22]. Together with the known early expansion of the lysosomal compartment within dying neurons in Alzheimer's disease [3] and proliferation of lysosomes after cathepsin inhibition in hippocampal slice culture [30] these data provided a rationale for testing whether similar lysosomal abnormalities occurred in the cathepsin D null flies. Accordingly, whole mount fly brains were dissected, stained with lysotracker, and examined by confocal microscopy for lysosomal abnormalities. Compared to control brains, cathepsin D null flies displayed a significant enlargement of individual lysosomes, and proliferation of the lysosomal compartment, with single cells sometimes containing multiple large lysosomes (Figure 6C–6F). To further examine the lysosomal compartment in our cathepsin D mutant animals, we used quantitative Western blot analysis to assess the levels of a GFP-tagged intralysosomal protease, cysteine protease-1 (Cp-1). We found a marked increase in the levels of Cp-1 in cathepsin D null animals compared to controls (Figure 6B). These data, together with our prior analysis of cathepsin D mutant flies [22], demonstrate that removing cathepsin D leads to striking lysosomal abnormalities that plausibly underlie the caspase activation induced by removal of cathepsin D.

### Loss of cathepsin D results in caspase activation and accumulation of caspase-cleaved tau in cathepsin D mutant mice and sheep

**Figure 7:**
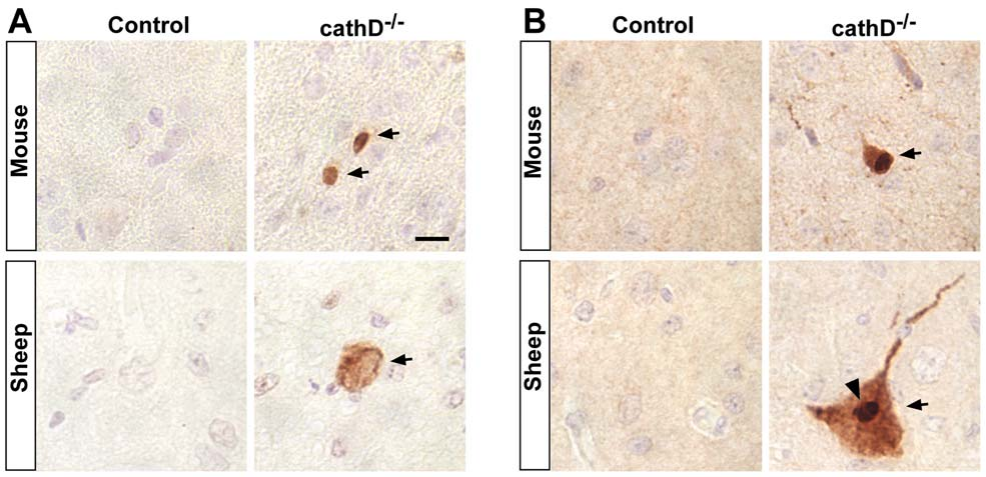
Caspase activation and truncation of tau in mammalian models of cathepsin D deficiency. (A) Caspase activation is present in the cortex of mice (upper panels, arrows) and sheep (lower panels, arrow) lacking cathepsin D, but not in controls. (B) C-terminally truncated tau as detected with the specific antibody tau-C3 is also found in cathepsin D mutant mice (upper panels, arrow) and sheep (lower panels, arrow), but not in controls. Arrowhead indicates dense staining, consistent with inclusion formation. Scale bar (A) is 10 µm and applies to all panels.

Loss of cathepsin D results in an aggressive form of the lysosomal storage disease neuronal ceroid lipofuscinosis in human patients [13], [14]. Similarly, both sheep and mice lacking cathepsin D activity recapitulate the aggressive neurodegeneration in the central nervous system, marked expansion of lysosomal compartment and autofluorescent lysosomal storage material found in the human disease [42], [43]. We thus asked whether loss of cathepsin D in these models leads to caspase activation, as predicted by our findings in the fly model. We found that in both mouse and sheep models of cathepsin D deficiency, there were activated caspase-3 immunopositive neurons throughout the cortex (Figure 7A, arrows). Activated caspase was not seen in cortical sections from control animals. Importantly, activated caspase was accompanied by C-terminally truncated tau, as detected by immunostaining with the tau-C3 monoclonal antibody (Figure 7B, arrows). Some tau-C3-positive cells displayed dense, punctate staining consistent with inclusion formation (Figure 7B, arrowhead). Thus, in mammalian models, just as in flies, removal of cathepsin D leads to caspase activation and cleavage of tau at Asp-421.

## Discussion

Almost two decades ago Cataldo and Nixon described early and striking lysosomal upregulation in susceptible and dying neurons in Alzheimer's disease [44]. Their data raised the possibility of an important lysosomal contribution to Alzheimer's disease and other neurodegenerative diseases. Indeed, the presence of an entire class of neurodegenerative diseases caused by lysosomal dysfunction, the lysosomal storage diseases, has emphasized the dependence of postmitotic neurons, as well as other cell types, on an intact lysosomal system [45], [46]. However, the precise mechanisms by which lysosomal abnormalities may contribute to neurodegeneration in Alzheimer's disease and related tauopathies have remained elusive. In particular, the impact of alterations in the lysosomal system described in dystrophic neurites on tau aggregated in somatodendritic compartments has not been clear. To further investigate the connection between the lysosomal system and Alzheimer's disease pathogenesis, we have used a genetically tractable *in vivo* model system. Our data support a contribution of lysosomal dysfunction to neurodegeneration in Alzheimer's disease and related tauopathies.

### Cathepsin D is an important modulator of tau-induced neurotoxicity in Alzheimer's disease and the fly tauopathy model

The early papers demonstrating lysosomal abnormalities in Alzheimer's disease brain demonstrated upregulation of cathepsin D mRNA and protein in dying neurons in the disease [2]. The connection between Alzheimer's disease and cathepsin D has also been made in multiple additional studies, including investigations of patient brain tissue, cerebrospinal fluid, fibroblasts, and genetic studies [9], [11], [12], [47], [48]. A number of genetic linkage studies have suggested a role for cathepsin D in the pathogenesis of Alzheimer's disease, although these results have not been replicated in all cohorts examined, including recent genome wide association studies [49], [50]. Nonetheless, a recent candidate gene analysis in a large population-based cohort study as well as a meta-analysis of previous studies supports the association of a *CTSD* variant and Alzheimer's disease risk [51].

The complete loss of cathepsin D in patients results in a devastating form of the lysosomal neurodegenerative disorder, neuronal ceroid lipofuscinosis [13], [14], [52]. Furthermore, cathepsin inhibition in hippocampal slice and cell culture studies has previously been shown to affect tau proteolysis and solubility [5], [30]. We therefore determined whether cathepsin D downregulation could influence tau-induced neurotoxicity in an animal model of Alzheimer's disease and related tauopathies. We found that removing cathepsin D substantially potentiated tau-induced neurotoxicity, strongly supporting a role for cathepsin D and the normal lysosomal system in maintaining the viability of postmitotic neurons. Removing cathepsin D genetically had no effect on neurotoxicity in a distinct *Drosophila* model of neurodegenerative disease in which the polyglutamine-expanded SCA3 protein is over-expressed, supporting specificity of the effect.

### Caspase-cleaved tau links lysosomal dysfunction to tau-induced neurotoxicity

A number of different hypotheses have related cathepsin dysregulation to Alzheimer's disease. *In vitro*, cathepsins can cleave proteins believed to be toxic in neurodegenerative diseases, including tau [8]. In our fly model, however, the marked increase in tau toxicity in a cathepsin D null genetic background was not accompanied by a change in tau levels (Figure 2). Tau phosphorylation is a well-described post-translational modification in tauopathies, including Alzheimer's disease, and plays a critical role in mediating tau neurotoxicity in *Drosophila* [27], [28], [29], but there was no change in tau phosphoepitope levels to explain the modulatory effect of cathepsin D (Figure 2).

We next directed our attention to levels of the C-terminally truncated, caspase-cleaved form of tau that is specifically identified by the tau-C3 monoclonal antibody. Truncated tau has been implicated in the pathogenesis of Alzheimer's disease and related tauopathies because the C-terminally cleaved protein is found in neurofibrillary lesions (neurofibrillary tangles and neuritic threads) in Alzheimer's disease and related tauopathies [31], [32], [33]. C-terminal truncation of tau also occurs in murine models of tauopathy, where caspase activation correlates with neurofibrillary tangle formation [53]. Further, C-terminally truncated tau has significant toxicity in cultured neurons [34], [35], [36]. We found that cathepsin D removal markedly increased levels of C-terminally truncated tau (Figure 3). Truncated tau appeared in a restricted number of neurons (Figure 3, Figure S3), making it difficult to evaluate the total cytosolic concentration of the C-terminally truncated species. Since neurons containing truncated tau also immunostained preferentially for the cell cycle marker PCNA (Figure 4B), it is possible that C-terminal truncation of tau is a relatively late event that leads to cell cycle activation and subsequent neuronal apoptosis. Importantly, tau-induced caspase activation co-immunolocalized with tau-C3, supporting a role for activated caspase in the generation of truncated tau in our *in vivo* system (Figure 4A). We further verified that tau^1–421^, when expressed in flies, was substantially more toxic and less soluble than wild-type tau. Utilizing the power of *Drosophila* genetics, we demonstrated that, unlike the case for wild-type tau, the toxicity induced by tau^1–421^ was not exacerbated by removal of cathepsin D (Figure 5B). This genetic result established a causal role for C-terminal truncation of tau in the exacerbation of tau neurotoxicity by removal of cathepsin D in our model system.

Lysosomal dysfunction offers an attractive link between cathepsin D depletion and caspase-dependent cleavage of tau for several reasons. First, abnormal expansion of the lysosomal system is well described in Alzheimer's disease brain, as noted above, and cathepsins are critical components of the lysosome. Second, loss of cathepsin D function in humans causes marked lysosomal proliferation, abnormal accumulation of lysosomal storage material and aggressive neurodegeneration in an infantile form of neuronal ceroid lipofuscinosis [13], [14], [52]. Third, lysosomal dysfunction is known to lead to abnormal lysosomal permeability, release of lysosomal contents and subsequent cytosolic activation of caspases [7]. Supporting this model, we demonstrate that loss of cathepsin D leads to abnormal intraneuronal lysosomal expansion in the adult fly brain and an increase in caspase activity (Figure 6). Interestingly, oxidative stress, a well-described trigger of increased lysosomal permeability and implicated both in Alzheimer's disease and in our *Drosophila* tauopathy model [17], may be a potential contributor to the marked increase in tau toxicity observed in the cathepsin D null background.

### Caspase-mediated tau cleavage and lysosomal dysfunction: unifying mechanisms of toxicity in diverse brain diseases?

A variety of neurodegenerative conditions have been associated with neurofibrillary tau pathology, ranging from repeated head trauma to abnormal prion protein accumulation [54]. Of particular relevance to the current study, tau aggregation is also an invariant pathology in certain lysosomal disorders, most notably Niemann Pick Type C. The prototypic “secondary” tauopathy is, of course, Alzheimer's disease in which abnormal amyloid precursor protein processing and beta-amyloid accumulation is considered the inciting event, and tau aggregation an essential downstream mediator of neurotoxicity [55]. The existence of primary tauopathies, particularly tauopathies caused by mutations in *MAPT* (FTDP-17) [56], [57], [58], make it improbable that tau abnormalities are bystander events in these multiple disorders that exhibit neurofibrillary pathology.

In Alzheimer's disease there is considerable experimental support for an important role of tau in the pathogenesis of the disease. For example, experiments in a variety of animal models have demonstrated synergistic effects of tau and beta-amyloid on protein aggregation and neurodegeneration [20], [59], [60]. In an elegant recent study, toxicity produced by expression of human amyloid precursor protein in a mouse model of Alzheimer's disease was completely blocked by removing endogenous mouse tau [61]. Importantly, the same study demonstrated that removing endogenous mouse tau also protected from excitotoxin-induced seizures, even in the absence of human amyloid precursor protein expression. These findings suggest that tau may play a toxic role even in the absence of fibrillary tau pathology.

A number of studies suggest that various upstream inciting neurotoxic factors promote neurotoxicity and neuronal cell loss via post-translational modifications of tau. While considerable attention has been directed to phosphorylation of tau, our data support a growing literature implicating C-terminally truncated tau as a critical downstream effector. Caspase cleavage of tau has been well documented in postmortem brain tissue from patients with Alzheimer's disease, and truncated tau shows significant toxicity in cultured cells. As noted above, in this study we confirm enhanced neurotoxicity of truncated tau in an animal model. Intriguingly, C-terminally truncated tau has also been reported in other tauopathies including Pick's disease, corticobasal degeneration and progressive supranuclear palsy [32], and even possibly in other neurodegenerative diseases, including Parkinson's disease and related synucleinopathies [62]. Together, these data suggest that C-terminally truncated tau may be an important effector of neurotoxicity in tauopathies and possibly other neurodegenerative conditions.

Like tau aggregation, lysosomal dysfunction is also feature of a diverse group of neurodegenerative diseases [6]. We have focused on the evidence for lysosomal dysfunction in Alzheimer's disease thus far in the current report, but lysosomal dysfunction has been suggested in other disorders, including Parkinson's disease, amyotrophic lateral sclerosis, prion diseases, and even in models of traumatic brain injury [6], [63]. Indeed, lysosomal storage diseases, in which severe and often early onset neurodegeneration is caused by mutations in lysosomal protein-encoding genes, suggest that postmitotic neurons are dependent on normal lysosomal function [1]. In this study we establish a link *in vivo*, in the setting of cathepsin D removal, between lysosomal dysfunction, caspase activation and tau truncation. We further suggest a similar relationship in mammalian systems because we show activated caspase and formation of caspase-cleaved tau in cathepsin D null mice and sheep, both well-established models of neuronal ceroid lipofuscinosis [42], [43].

In summary, the data outlined in this study have suggested causal connections *in vivo* between lysosomal dysfunction and neurodegeneration in a model of Alzheimer's disease and other tauopathies. Our data support a scenario in which normal levels of cathepsin D are required for proper lysosomal function, and in which caspase cleavage of tau is a a neurotoxic effector of lysosomal dysfunction in these diseases. A link between the lysosomal system and tau fragmentation has been supported by recent work from the Mandelkow laboratory as well [64]. We speculate that lysosomal dysfunction and subsequent caspase-mediated cleavage of tau may be common mechanisms of neurodegeneration in a diverse number of diseases, and attractive targets for therapeutic intervention.

## Materials and Methods

### Transgenic *Drosophila*

Flies were grown on standard cornmeal medium at 25°C. *UAS-tau^1–421^* flies were created by introducing a stop codon following residue 421 in the human wild-type tau cDNA. A 0 N-terminal insert, 4 microtubule binding repeat (0N4R) form of tau was used in all experiments. The tau^1–421^ mutant cDNA was cloned into the GAL4-responsive pUAST expression vector. Transgenic strains were created by embryo injection. Ten independent transgenic lines were obtained and analyzed. The *UAS-SCA3* [25], [26], *UAS-tau^WT^*, *UAS-tau^R406W^* [19], and *cathD^1^* mutant [22] stocks have been previously described. The panneural *elav-GAL4* driver was obtained from the Bloomington *Drosophila* Stock Center (Bloomington, IN) and was used in all experiments. The *Cp1-GFP* (ZCL2854) flies were obtained from W. Chia [65] via L. Cooley.

Lifespan analysis was performed as described previously [19]. Briefly, flies were aged in groups of no more than 25 flies per vial. Culture medium was changed every 2 days and the number of dead flies recorded. At least 300 flies were aged for each genotype.

RNA isolation, quantitative PCR, and quality controls were previously described [21]. Briefly, RNA was isolated from heads of flies aged to 1, 10, or 30 days, quantitative PCR was performed using the Comparative C_T_ method and GAPDH as reference gene. Approximately equal amplification efficiencies of target and reference gene were previously confirmed [21].

### Immunohistochemistry and histology

Adult flies or mouse or sheep brain tissue was fixed in formalin and embedded in paraffin. Brains from three *cathepsin D* knockout mice were examined (two postnatal day (P) 20, one P24), and from three controls (two P20, one P24). Tissue from three cathepsin D deficient sheep and two control sheep was examined. Sheep were one day old. For *Drosophila* tissue, serial frontal sections (4 µm) were made of the entire brain. To assess general brain morphology, vacuolar degeneration, and Kenyon cell density, sections were stained with hematoxylin and eosin. Vacuolar degeneration was assessed by counting vacuoles larger than 3 µm in at least six hemibrains per genotype. To evaluate Kenyon cell loss in the ataxin 3 model, the number of cells in a defined 35 mm area in well-oriented frontal sections was counted. At least six hemibrains were counted per genotype.

For immunostaining, antigen retrieval was performed by microwaving in sodium citrate (pH 6.0) for 15 minutes. The primary antibodies used were tau-C3 (1∶1,000, Biosource), PCNA (1∶100, Biomeda), and activated Caspase-3 (1∶250, Trevigen). Secondary detection was performed with an avidin-biotin-peroxidase complex (ABC) method (Vector Laboratories) or with secondary antibodies coupled to Alexa Fluor 488 or Alexa Fluor 555. For quantitation of PCNA staining, the number of PCNA-positive foci was counted in at least 6 brains per genotype.

### TUNEL analysis

Brain sections from 10-day-old flies were stained using the TUNEL assay (TdT FragEL DNA fragmentation kit; EMD Biosciences) and the avidin-biotin-peroxidase complex (ABC) detection method (Vectastain ABC Kit; VectorLaboratories). Quantification of TUNEL-positive foci was performed on at least six hemibrains (Figure 1) or six whole brains (Figure 5) per genotype.

### Western blot and tau solubility analysis

Heads from 10 day-old-adult *Drosophila* were homogenized in Laemmli buffer, resolved by SDS-PAGE and immunoblotted as indicated. The primary antibodies used were rabbit polyclonal anti-tau (1∶10^6^, Dako), tau-C3 (1∶1,000, Biosource), anti-actin (1∶50,000, Sigma-Aldrich), AT8 (1∶50,000, Pierce Biotechnology), TG3 (1∶1,000, P. Davies, Albert Einstein College of Medicine, Bronx, New York, USA), PHF1 (1∶50,000, P. Davies), AT180 (1∶2,000, Pierce Biotechnology), and AT270 (1∶10,000, Pierce Biotechnology). The polyclonal antibody against tau from Dako recognizes sequences near the microtubule binding domain and was used to assess levels of total tau in Figure 2, Figure 3, Figure 5, Figure S4. Secondary antibodies conjugated to HRP (SouthernBiotech) were used at 1∶50,000 dilution, and signal detection was performed with chemiluminescence (Pierce Biotechnology). All Western blots shown (Figure 2, Figure 3, Figure 5, Figure 6) were repeated at least three times with similar results.

Nonionic detergent-soluble and -insoluble protein fractions were prepared by homogenization of fly heads in TNE buffer (10 mM Tris-HCl, pH 7.4; 150 mM NaCl; 5 mM EDTA) containing protease inhibitors (Roche Complete Protease Inhibitor Cocktail) and detergents (0.5% Nonidet P-40), as previously described [66]. The homogenate was centrifuged (5 min at 100,000×g), and the resulting pellet and supernatant fractions were collected. The pellet was washed once in TNE containing nonionic detergents, and the resulting pellet (nonionic detergent-insoluble) was solubilized in TNE buffer containing 1% SDS. Equivalent amounts of soluble and insoluble material from tau^WT^ and tau^1–421^ homogenates were loaded to ensure accurate comparisons between the two genotypes.

### Lysosome analysis

In order to visualize lysosomal morphology, fresh whole mount brains from 10 day-old flies were incubated with 100 µM lysotracker for 5 minutes, mounted in PBS, and analyzed with a Zeiss laser-scanning confocal microscope.

## Supporting Information

Figure S1 Cathepsin D is overexpressed in tau-transgenic flies at early and advanced disease-stages. Relative cathepsin D mRNA abundance is 1.6-fold and 2-fold elevated in 10-day-old and 30-day-old tau transgenics compared to age-matched control animals using quantitative PCR (***P = 0.005 and **P = 0.037), consistent with our microarray screen [21].
(0.11 MB TIF)

Figure S2 No change in the neurotoxicity of mutant ataxin 3 in the absence of cathepsin D. Expression of expanded ataxin 3 (MJD) produces significant loss of Kenyon cells, and that loss is not exacerbated by removing cathepsin D. Flies are 10 days old. Control is *elav-GAL4/+*.
(0.28 MB TIF)

Figure S3 Specificity of tau-C3 in immunofluorescence analysis. (A) There is no tau-C3 immunoreactivity in control flies (genotype: *elav-GAL4/+*). (B) Immunoreactivity for tau-C3 is present in selected neurons in flies expressing tau (genotype: *elav-GAL4/+;UAS-tau-R406W/+*). (C) Widespread tau-C3 immunoreactivity is present in brains of flies expressing C-terminally truncated tau (genotype: *elav-GAL4/+;UAS-tau-1-421/+*). Sections were cut, processed, immunostained, and imaged in parallel. Imaging conditions (including time of camera exposure) were held constant for panels A and B, but a weaker exposure of C is shown to allow appreciation of cellar detail. Flies are 10 days old.
(1.27 MB TIF)

Figure S4 Specificity of tau-C3 immunostaining in Western blotting analysis. Homogenates from control flies (genotype: *elav-GAL4/+*) and flies expressing C-terminally truncated tau (genotype: *elav-GAL4/+;UAS-tau-1-421/+*) were run on the same gel in the order indicated. The gel was then cut in half as indicated by the dotted line and hybridized with the tau-C3 monoclonal antibody (left) or a polyclonal antibody recognizing tau (anti-tau, Dako, right). The blot was reprobed with an antibody recognizing actin to evaluate protein loading. Flies are 10 days old.
(0.12 MB TIF)

Figure S5 Soluble fractions from flies expressing wild-type tau and C-terminally truncated tau. The entire molecular weight range, together with molecular weight markers in KDa is shown for the blot in Figure 5C.
(0.07 MB TIF)

Figure S6 Insoluble fractions from flies expressing wild-type tau and C-terminally truncated tau. The entire molecular weight range, together with molecular weight markers in KDa is shown for the blot in Figure 5D.
(0.19 MB TIF)
