# Supplementary figures and images for: Lysosomal Dysfunction Promotes Cleavage and Neurotoxicity of Tau In Vivo
Source: PLoS Genet. 2010 Jul 15;6(7):e1001026. doi: 10.1371/journal.pgen.1001026 (PMC2904797; doi:10.1371/journal.pgen.1001026)

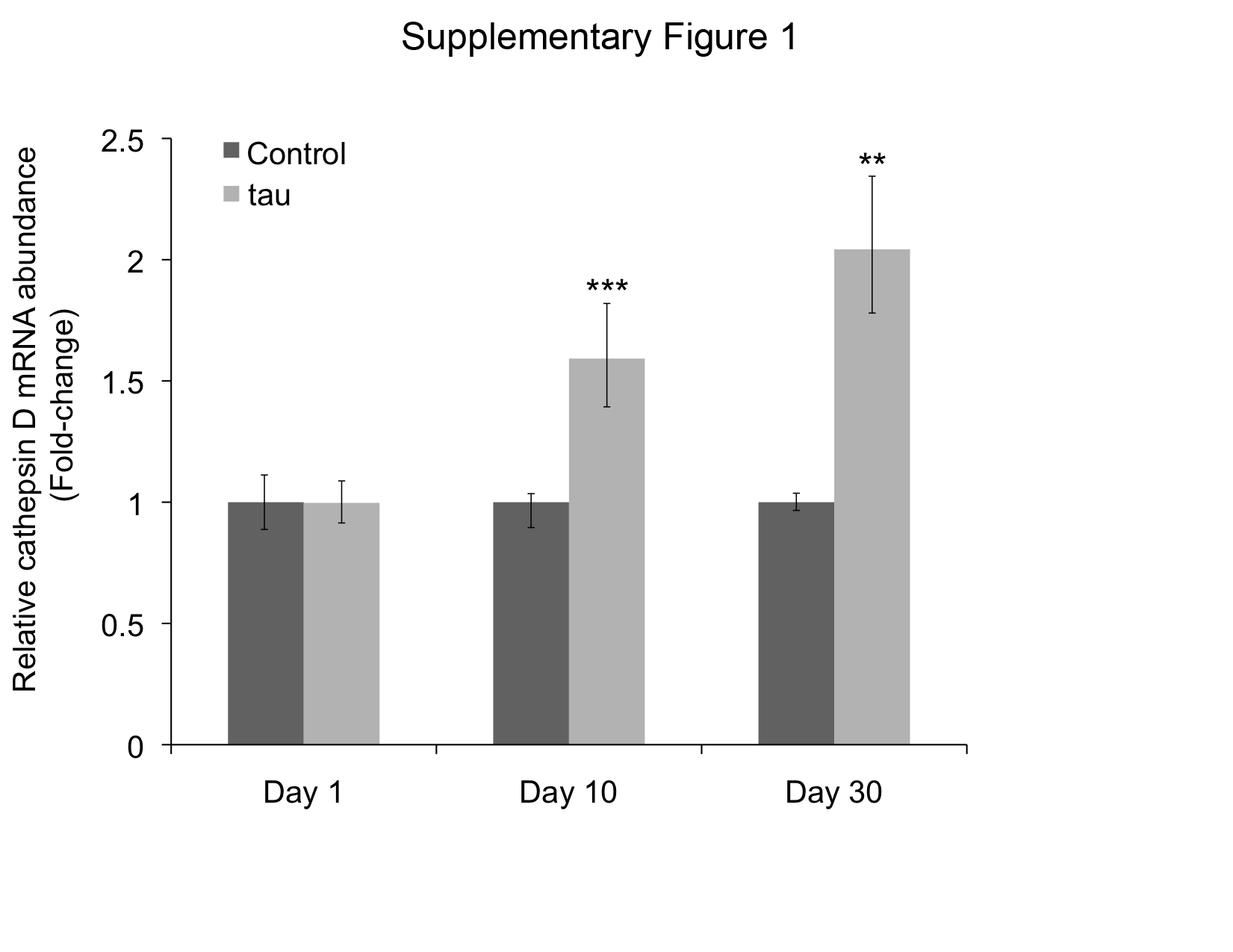

Supplement: Figure S1 — Cathepsin D is overexpressed in tau-transgenic flies at early and advanced disease-stages. Relative cathepsin D mRNA abundance is 1.6-fold and 2-fold elevated in 10-day-old and 30-day-old tau transgenics compared to age-matched control animals using quantitative PCR (***P = 0.005 and **P = 0.037), consistent with our microarray screen [21]. (0.11 MB TIF) [file pgen.1001026.s001.tif]

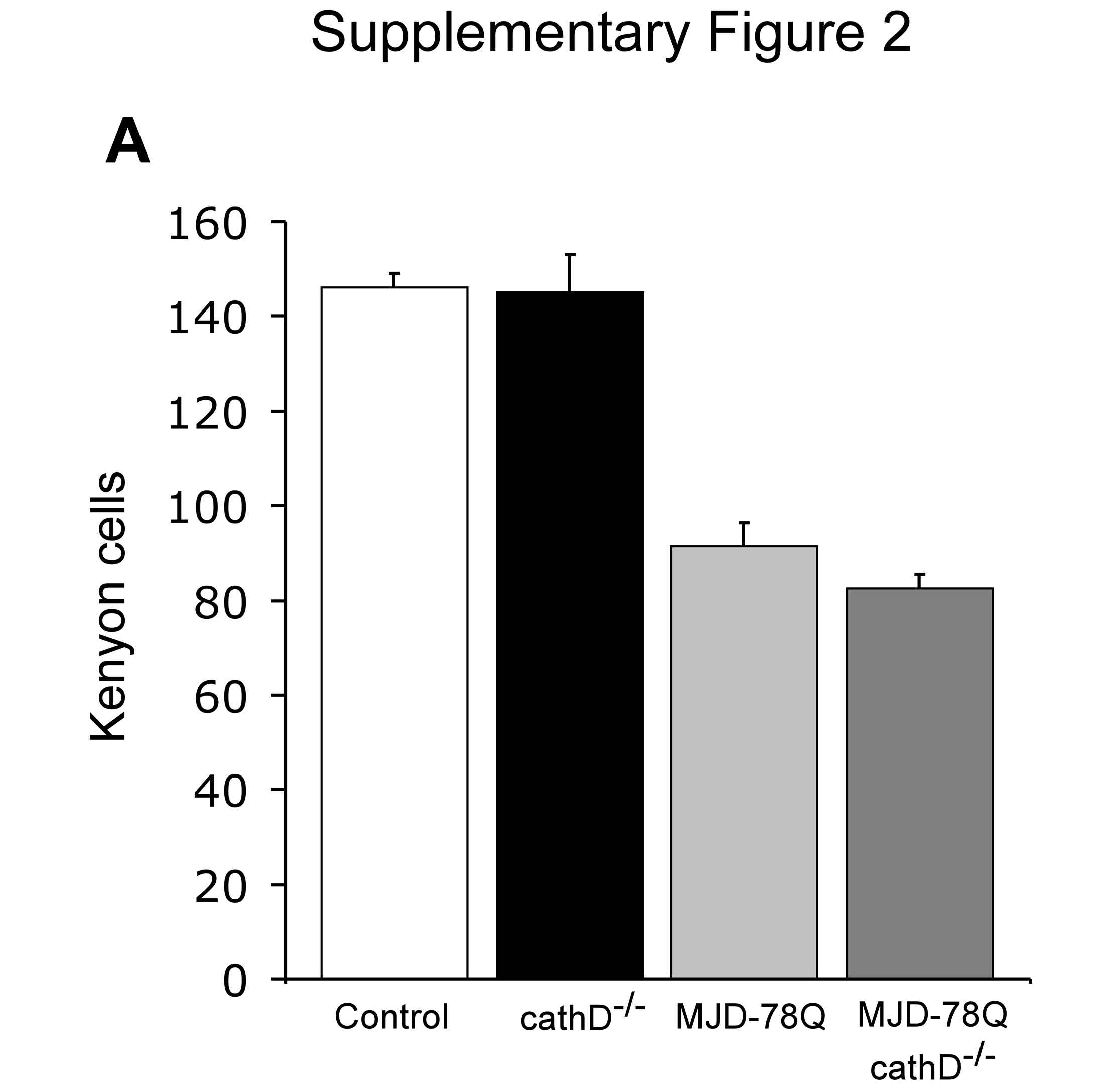

Supplement: Figure S2 — No change in the neurotoxicity of mutant ataxin 3 in the absence of cathepsin D. Expression of expanded ataxin 3 (MJD) produces significant loss of Kenyon cells, and that loss is not exacerbated by removing cathepsin D. Flies are 10 days old. Control is elav-GAL4/+. (0.28 MB TIF) [file pgen.1001026.s002.tif]

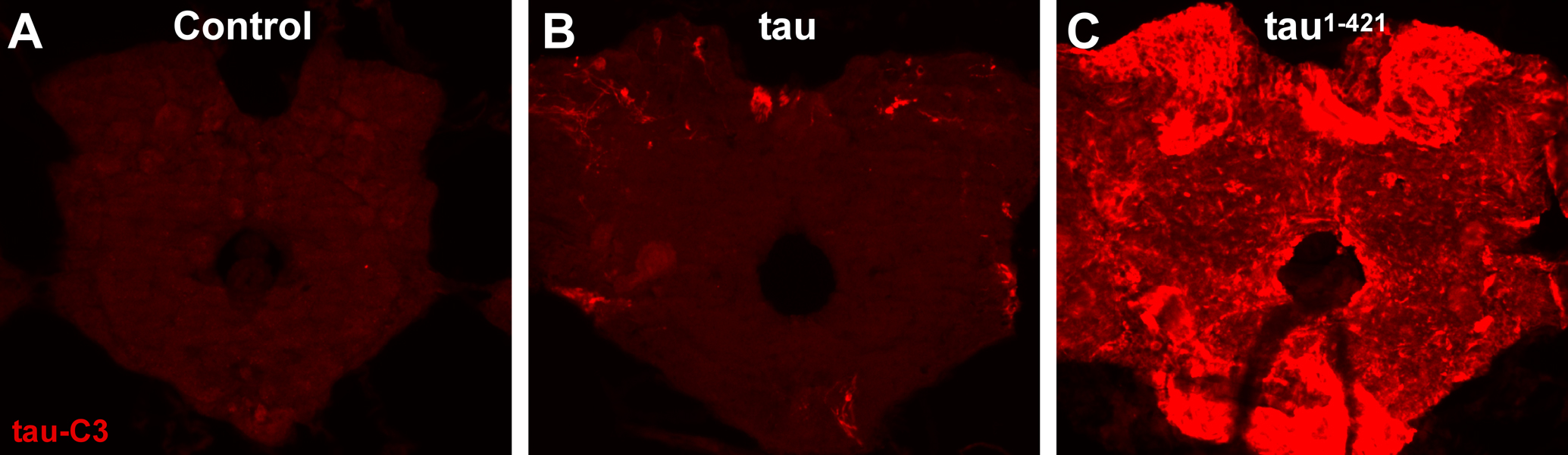

Supplement: Figure S3 — Specificity of tau-C3 in immunofluorescence analysis. (A) There is no tau-C3 immunoreactivity in control flies (genotype: elav-GAL4/+). (B) Immunoreactivity for tau-C3 is present in selected neurons in flies expressing tau (genotype: elav-GAL4/+;UAS-tau-R406W/+). (C) Widespread tau-C3 immunoreactivity is present in brains of flies expressing C-terminally truncated tau (genotype: elav-GAL4/+;UAS-tau-1-421/+). Sections were cut, processed, immunostained, and imaged in parallel. Imaging conditions (including time of camera exposure) were held constant for panels A and B, but a weaker exposure of C is shown to allow appreciation of cellar detail. Flies are 10 days old. (1.27 MB TIF) [file pgen.1001026.s003.tif]

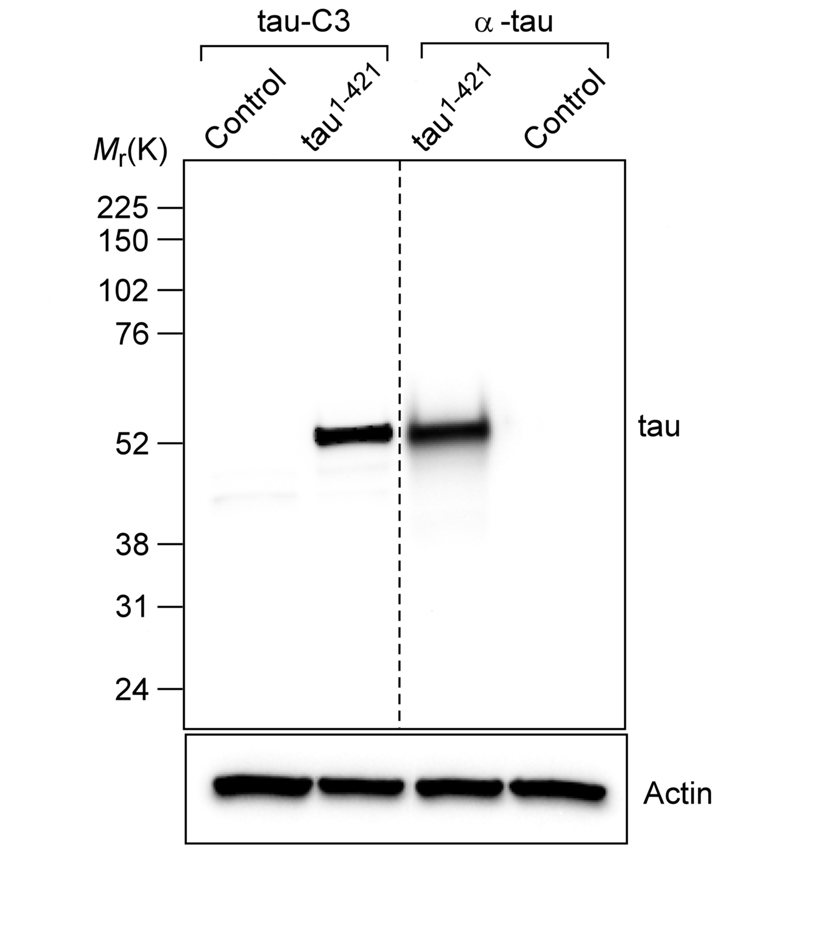

Supplement: Figure S4 — Specificity of tau-C3 immunostaining in Western blotting analysis. Homogenates from control flies (genotype: elav-GAL4/+) and flies expressing C-terminally truncated tau (genotype: elav-GAL4/+;UAS-tau-1-421/+) were run on the same gel in the order indicated. The gel was then cut in half as indicated by the dotted line and hybridized with the tau-C3 monoclonal antibody (left) or a polyclonal antibody recognizing tau (anti-tau, Dako, right). The blot was reprobed with an antibody recognizing actin to evaluate protein loading. Flies are 10 days old. (0.12 MB TIF) [file pgen.1001026.s004.tif]

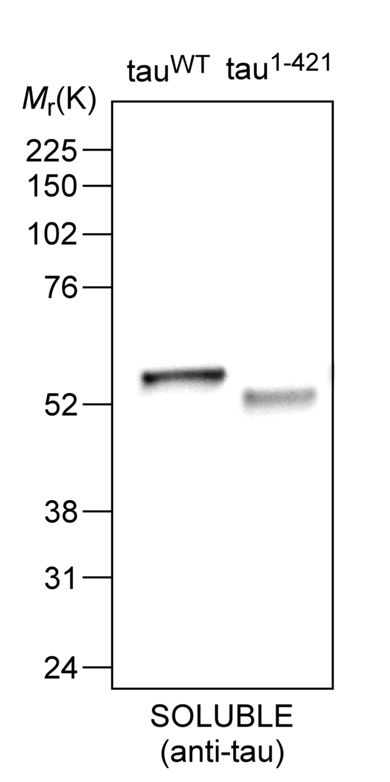

Supplement: Figure S5 — Soluble fractions from flies expressing wild-type tau and C-terminally truncated tau. The entire molecular weight range, together with molecular weight markers in KDa is shown for the blot in Figure 5C. (0.07 MB TIF) [file pgen.1001026.s005.tif]

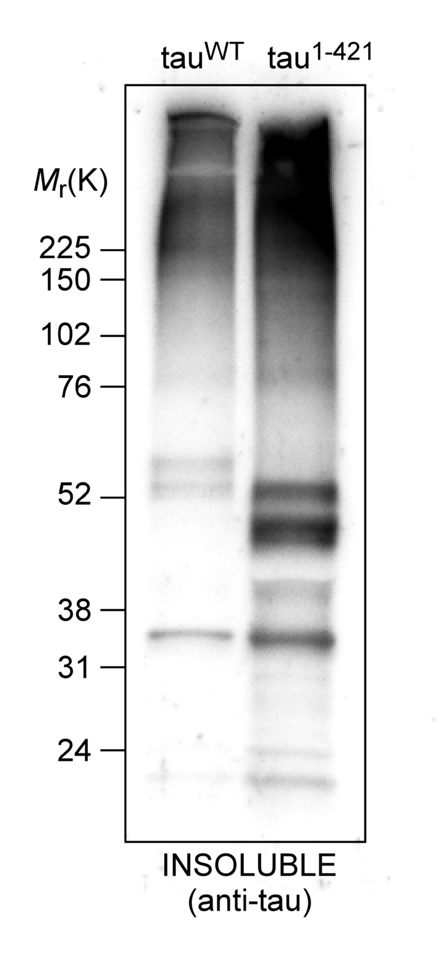

Supplement: Figure S6 — Insoluble fractions from flies expressing wild-type tau and C-terminally truncated tau. The entire molecular weight range, together with molecular weight markers in KDa is shown for the blot in Figure 5D. (0.19 MB TIF) [file pgen.1001026.s006.tif]
